# Knowledge, Attitudes, Practices, and Risk Perception of MERS-CoV and Other Coronaviruses Among Health Care Workers in Northern Kenya

**DOI:** 10.4269/ajtmh.25-0585

**Published:** 2026-05-12

**Authors:** Cynthia Ombok, Andrew Karani, Carolyne Nasimiyu, Moshe Alando, Ali Badha, Boru Ali, Millicent Minayo, Boru Wato, Elizabeth Mungai, Raymond Odinoh, Boku Bodha, M. Kariuki Njenga, Isaac Ngere

**Affiliations:** ^1^ Centers for Research in Emerging Infectious Diseases–East and Central Africa, Nairobi, Kenya;; ^2^ Department of Medical Microbiology, University of Nairobi, Nairobi, Kenya;; ^3^ Department of Health, Isiolo County, Isiolo, Kenya;; ^4^ Department of Veterinary Services, Marsabit County, Marsabit, Kenya;; ^5^ Center for Global Health Research, Kenya Medical Research Institute, Kisumu, Kenya;; ^6^ Paul Allen School for Global Health, Washington State University, Pullman, Washington, USA

## Abstract

Middle East respiratory syndrome coronavirus (MERS-CoV) remains a global health threat, with frequent hospital outbreaks in the Middle East and Asia. Transmission occurs through contact with dromedary camels, and spillover risk in Northern Kenya is significant due to high camel exposure and growing human mobility. We assessed knowledge, attitudes, practices, and risk perception (KAP-RP) regarding MERS-CoV and other coronaviruses among health care workers (HCWs) in Isiolo and Marsabit counties. A cross-sectional survey was conducted between December 2023 and January 2024. Using two-stage stratified random sampling, HCWs from public health facilities were recruited, covering clinical and nonclinical cadres. Data were collected using a structured, pretested questionnaire on KAP-RP. Scores were classified as sufficient or insufficient. Analyses included descriptive statistics, logistic regression, and Pearson correlations. Of 847 HCWs enrolled (36.4% Isiolo, 63.6% Marsabit), the majority were age 25–34 years, with a male:female ratio of 1.3:1. Clinical cadres made up 56.2%, and 77% worked in rural facilities. Only 28.1% demonstrated sufficient knowledge, 74.7% showed favorable attitudes, 25.1% adhered adequately to preventive practices, and 26.7% had high risk perception. PPE use was reported by 85.5%, but only 49.2% wore it consistently, and 33.3% changed it as required. Knowledge was higher among males, older HCWs, and Isiolo residents. Correlation analysis showed a weak positive association between knowledge and practice, a negative association with attitude, and minimal links with risk perception. Substantial knowledge gaps, inconsistent practices, and low risk perception persist, highlighting the need for context-specific, behavior-focused training to strengthen preparedness in zoonotic hot spots.

## INTRODUCTION

Of the seven human coronaviruses, three are associated with severe and potentially fatal human health outcomes, including severe acute respiratory syndrome coronavirus (SARS), which emerged in late 2002 and was formally recognized in 2003; Middle East respiratory syndrome coronavirus (MERS-CoV), which emerged in 2012; and SARS-CoV-2, which emerged in 2019.^1^ Of the three, only MERS-CoV has a domestic animal reservoir—the dromedary camel (*Camelus dromedarius*) that is native to most parts of the Horn of Africa (HOA), where over 65% of this animal species reside.^2^ Since its emergence over a decade ago, MERS-CoV has caused >2,500 cases in 27 countries, with most of the cases arising from health care settings in the Kingdom of Saudi Arabia.^3^

MERS-CoV is thought to freely circulate in the camel population, with animal-to-animal transmission driven mainly by husbandry practices and the specific MERS-CoV virus strain.^4^^,^^5^ Of the three clades of MERS-CoV described so far, clade C is prevalent in African camels and is thought to cause widespread seasonal camel infections, with very limited burden of human disease.^6^^,^^7^ Human-to-human transmission is through respiratory droplet pathways and by person-to-person contact. Sick individuals shed the viruses when they cough, sneeze, or spit sputum/saliva without observing the required infection prevention measures.^3^ In the case of MERS-CoV, occupational exposure primarily increases the risk of zoonotic transmission from infected dromedary camels, particularly among camel herders and camel abattoir workers. Limited human-to-human transmission has been documented, most often in health care settings, placing health care workers (HCWs) attending patients with respiratory illness in camel-keeping areas at additional risk when infection prevention measures are inadequate.^3^^,^^6^^,^^8^

Serological test results and molecular evidence have pointed at low-level, unsustained MERS-CoV infection in people in northern Kenya.^6^^,^^7^^,^^9^^,^^10^ Recent assessments by the Food and Agriculture Organization of the United Nations (FAO) indicate that there is a moderate risk of introduction of more severe strains of MERS-CoV (Clade B) in the HOA region, given the limited surveillance activities targeting MERS-CoV in these regions.^11^ HCWs play a central frontline role in health facility disease detection, response, and prevention. Their knowledge of the disease, attitudes, and practices regarding MERS-CoV and other respiratory illness are key to ensuring that potential cases are identified early and isolated for the required treatments and to limiting the spread of infections in the health care setting.

Studies have highlighted limited availability of data on HCWs’ understanding and perception of MERS-CoV and related zoonotic coronaviruses in Africa.^12^^,^^13^ Even fewer studies are conducted among HCWs in arid and semiarid regions of the HOA where camel–human interactions are common.^14^^–^^16^ Assessment of HCWs’ knowledge and practices regarding MERS-CoV and other coronaviruses is necessary to establish the baselines and inform training needs and infection-prevention policies. In Kenya, although three confirmed human cases of MERS-CoV have been reported,^7^ no formal MERS-CoV–specific training or awareness campaigns had been conducted among HCWs at the time of this study. Consequently, expectations of HCW knowledge must be interpreted within this context. Assessment of HCWs’ knowledge, attitudes, practices, and risk perception regarding MERS-CoV and other coronaviruses is therefore necessary to establish baseline preparedness and to inform future training needs and infection prevention policies. Since 2018, we have conducted longitudinal studies in northern Kenya across community settings, slaughterhouses, and health facilities to investigate MERS-CoV transmission dynamics. Our previous work has documented widespread MERS-CoV infection among camels, with serological and molecular evidence indicating sporadic zoonotic transmission to individuals with occupational exposure to camels.^7^^,^^10^ The primary objective of this study was to assess the knowledge, attitudes, practices (KAP), and risk perception of MERS-CoV and other coronaviruses among HCWs in northern Kenya.

## MATERIALS AND METHODS

### Study site.

This cross-sectional study was conducted among HCWs across all operational health facilities, including small-volume health facilities (dispensaries and health centers) and referral health facilities (subcounty and county referral hospitals), in Isiolo and Marsabit counties in northern Kenya between December 2023 and January 2024. The two counties are predominantly inhabited by nomadic pastoralists whose livelihoods depend on camel herding, necessitating frequent migration in search of pasture and water.

### Sample size, participant selection, and consent.

**Figure 1.**
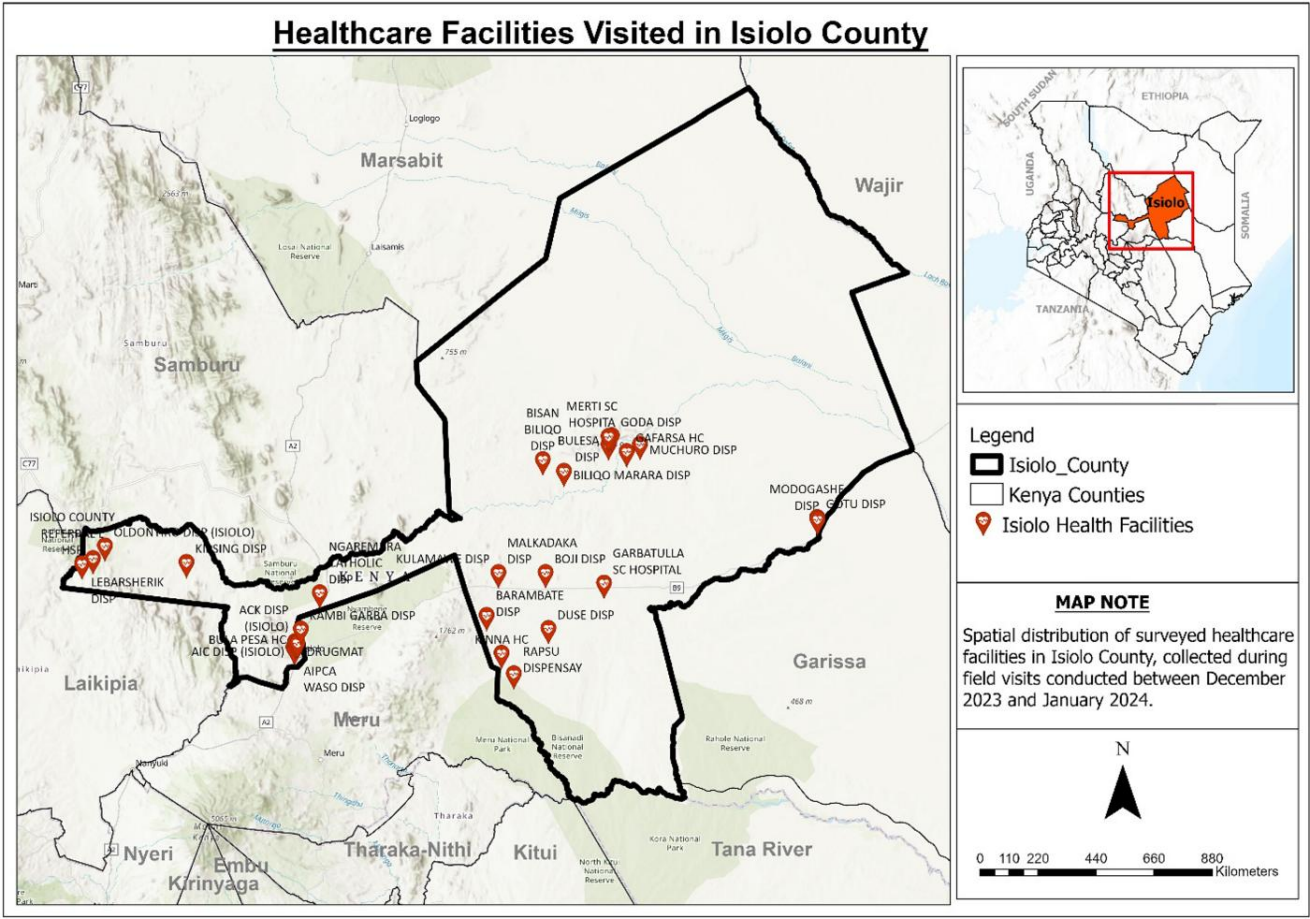
Map showing locations of health care facilities visited during the study in Isiolo County.

**Figure 2.**
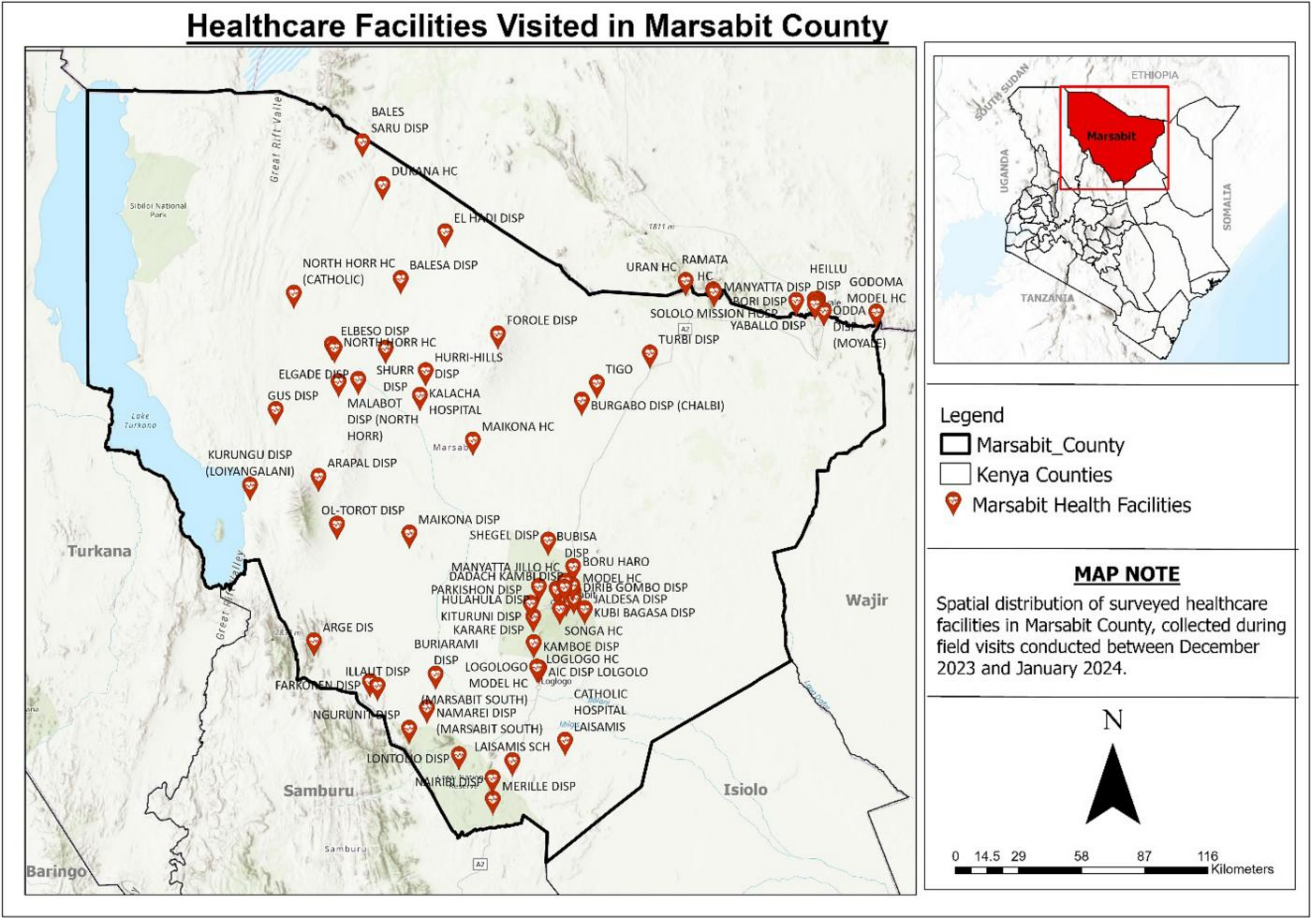
Map showing locations of health care facilities visited during the study in Marsabit County.

The KAP study was conducted as part of a MERS-CoV sero-survey among HCWs; hence the sample size was powered to the parent study. The sample size per county was estimated to range between 300 and 550 HCWs, using a standard formula for sample size calculation for proportions.^17^ This estimation assumed a 2% to 3% seroprevalence of MERS-CoV IgG, based on prior studies from Africa,^18^ and included a 5% margin to account for potential nonresponse. A comprehensive census of all operational health facilities in each county was developed, and facilities were randomly selected to ensure geographic representation across administrative wards and proportional inclusion of each level of care: county hospitals (Level 4 and 5), health centers (Level 3), and dispensaries (Level 2) (Figures 1 and 2). A two-stage stratified random sampling approach was used. In the first stage (strata 1), administrative subcounties were randomly selected, followed by the random selection of health facilities within those subcounties. Within selected facilities (strata 2), the number of HCWs to be sampled was determined using probability proportional to size (PPS) sampling, based on patient workload data from the preceding 12 months. At each health facility, all HCWs present and on duty on the day of data collection were enumerated, and the desired number of staff was randomly selected from the list, using a random number generator phone application for participation, regardless of cadre. The cadres included clinical personnel (such as medical doctors, clinical officers, nurses, and laboratory technologists) and nonclinical staff, such as records officers, nutritionists, social workers, cleaners, laundry staff, mortuary attendants, and administrators. To ensure adequate representation of the nonclinical cadres, staff from specific sections such as cleaning, mortuary, and laundry were specifically targeted. Each selected staff was consented using a paper consent form prior to data collection.

### Data collection.

The study questionnaire comprised five main sections. The first section collected demographic information, including participants’ age, gender, professional cadre, years of experience, primary roles, and departmental affiliation. The second section assessed participants’ knowledge and awareness of MERS-CoV, focusing on awareness of the virus, modes of transmission, clinical signs and symptoms, treatment options, and preventive measures. The third section explored attitudes toward MERS-CoV and COVID-19, as well as health-seeking behaviors related to the two viruses and influenza-like illnesses. The fourth section explored common infection prevention and control practices around their workplaces, e.g., use of personal protective equipment, whereas the last section explored the risk perceptions about MERS-CoV, coronaviruses, and other respiratory infections. Trained research assistants administered the questionnaire using REDCap^®^ data collection platform, following a pretesting exercise. Data collection was conducted on weekdays (Monday through Friday) between 0800 and 1700 hours.

### Scoring and evaluation of knowledge, attitudes, practices, and risk perception.

Participants’ responses were scored using a structured framework adapted from previously used KAP-RP tools. Each domain—knowledge, attitude, practice, and risk perception—was evaluated on a scale of 0 to 100, with specific thresholds used to classify responses as either sufficient or insufficient.

The knowledge domain comprised eight items assessing awareness and understanding of MERS-CoV and other coronaviruses. To reflect the importance of these foundational concepts, three questions in this section were assigned a higher weight of 20 points each. These questions addressed awareness of MERS-CoV and other coronaviruses, recognition of key clinical signs and symptoms, and knowledge that handwashing helps prevent the transmission of respiratory diseases. The remaining five knowledge questions, which covered transmission routes, treatment, prevention, susceptible populations, and postvaccination vulnerability, were each allocated 8 points. This scoring structure yielded a composite maximum score of 100%. Participants scoring 60% or more were classified as having sufficient knowledge, whereas those scoring below that threshold were considered to have insufficient knowledge.

Attitudes were assessed using two dichotomous items: concern about the risk of contracting MERS-CoV (with the correct response being “Yes”) and concern about contracting a coronavirus (correct response: “No”). Each correct answer was scored at 100%. However, for the overall attitude to be categorized as sufficient, both items needed to be answered correctly. This domain was treated as binary and was not summed as a continuous score.

Risk perception was assessed using three questions, each assigned equal weight (33.3 points), resulting in a total possible score of 99.9%, which was rounded to 100% for interpretation. A threshold of 66.6% was used to categorize high versus low risk perception.

The practice domain included eight items evaluating HCWs’ infection prevention and control behaviors. Three questions relating to consistent PPE use, appropriate hand hygiene during critical moments, and management of patients when PPE was unavailable were designated as high priority and assigned 20 points each. The remaining five questions, which addressed PPE change frequency, disposal practices, and other routine IPC measures, were weighed at 8 points each, yielding a maximum total of 100%. Participants scoring 60% or above were considered to have sufficient practice, whereas those with lower scores were categorized as insufficient.

## STATISTICAL ANALYSES

Data were analyzed using R software v. 4.3.2. (R Foundation for Statistical Computing) and using *stats*,* dplyr*,* tidyverse*,* and gtsummary* packages. Descriptive statistics were used to summarize the sociodemographic characteristics of the participants. All the categorical variables were presented as frequencies and percentages. The KAP-RP scores were computed from responses to relevant questionnaire items and categorized as “sufficient” or “insufficient,” based on predetermined scoring thresholds. Group comparisons of these outcomes across demographic variables were conducted using bivariate logistic regression, with odds ratios (ORs), 95% CIs, and corresponding *P*-values reported.

To assess the strength and direction of associations between the continuous KAP and risk perception scores, Pearson’s correlation coefficients were calculated. Correlations were considered significant at a two-tailed *P* <0.05. Confidence intervals for correlation coefficients were computed to provide estimates of precision.

## RESULTS

A total of 847 HCWs were enrolled, where Marsabit county had 539 (63.6%). There were more males, 474 (56.0%), than females, and most of the participants were 25–34 years of age (48.3%). Clinical cadres were 476 (56.2%), with the majority of the participants, 652 (77.0%), working in rural facilities. Table 1 shows the sociodemographic breakdown of participants.

**Table 1:** Demographic and socioeconomic characteristics of health care workers included in the KAP-RP study in northern Kenya

Characteristics	Overall (*N* = 847)		Isiolo, *n* = 308		Marsabit, *n* = 539	
	*N*	%	*n*	%	*n*	%
Sex						
Female	373	44.0	126	40.9	247	45.8
Male	474	56.0	182	59.1	292	54.2
Age categories (years)						
18–24	96	11.3	58	18.8	38	7.1
25–34	409	48.3	124	40.3	285	52.9
35–44	185	21.8	69	22.4	116	21.5
45–54	109	12.9	39	12.7	70	13.0
≥55	48	5.7	18	5.8	30	5.6
HCWs cadres						
Nonclinical*	371	43.8	117	38.0	254	47.1
Clinical^†^	476	56.2	191	62.0	285	52.9
Facility in charge(s)						
No	773	91.6	282	91.6	491	91.1
Yes	69	7.8	24	7.8	45	8.3
Missing	5	0.6	2	0.6	3	0.6
Region						
Rural^‡^	652	77.0	176	57.1	476	88.3
Urban^§^	195	23.0	132	42.9	63	11.7

KAP-RP = Knowledge, Attitudes, Practices, and Risk Perception.

* Nonclinical cadres included staff working in accounts, hospital board members, causality, link assistant, manager, storekeeper, administrative, administrator, cleaner, clerk, cook, data, driver, front office/customer care/secretary, gardener, grounds, health, health assistant, housekeeper, human resource, information, communication and technology clerk, interpreter, laundry, link assistants, maintenance personnel, medical engineer, mentor, mortuary, public, record’s officer, records officer, renal unit, security, social, social worker.

^†^ Clinical cadres included clinical officers, community health volunteers, dentists, doctors (consultant, medical officer), intern, laboratory technologist, nurse, nurse aide / nurse assistant, nutritionist, occupational, orthopedic, pharmacist/pharmacy technologist, physiotherapist, sonographer, student, theater.

^‡^ Urban health facilities: Marsabit County Referral Hospital, Moyale Sub-County Referral Hospital, and Isiolo County Referral and Teaching Hospital.

^§^ Rural: Nonreferral health facilities.

Table 2 breaks down the results of the participants’ responses in the KAP-RP study. Only 30.8% of respondents had prior awareness of MERS-CoV. Among those who were aware, recognition of key respiratory symptoms was high (90.4%), and 73.6% correctly identified preventive measures, although knowledge of treatment options (40.6%) and transmission routes (51.7%) was more limited. Awareness of hand hygiene as a general preventive measure against respiratory infections was high overall (88.4%) and was assessed among all respondents, not only those who had prior awareness of MERS-CoV.

**Table 2:** Participants’ responses to the KAP-RP questions

Question	*N*	Expected Response/Correct Answer	Respondents with the Correct Answer		Respondents with the Incorrect Answer		Mean Percent Score (SD)
			*n*	%	*n*	%	
Knowledge questions			*n*	%	*n*	%	
Have you ever heard of MERS-CoV?	847	Yes	261	30.8	586	69.2	41.1 (29.1)
Do you know of any other coronavirus?	586	Yes	464	79.2	122	20.8	
How does someone get MERS-CoV infection?	261	Aerosol, contact with body fluids, or related answers.	135	51.7	126	48.3	
What are the signs/symptoms of MERS-CoV?	261	Fever, cough, sore throat, running nose, body malaise, headache, chest congestion, DIB (at least 3 correct answers)	236	90.4	25	9.6	
How is MERS-CoV treated?	261	Use of antibiotics, supportive treatment, or responses in that line	106	40.6	155	59.4	
How is MERS-CoV prevented?	261	Avoiding sick animals, use of PPE, avoiding sick patients, vaccination, or other responses in that line (at least 1 correct answer)	192	73.6	69	26.4	
Who can get infected by MERS-CoV?	261	The elderly, adults or youth, and children.	234	89.7	27	10.3	
Can you get MERS-CoV after getting a COVID-19 vaccination?	261	Yes	179	68.6	82	31.4	
Can hand washing help prevent the spread of respiratory diseases?	847	Yes	749	88.4	98	11.6	
Attitude questions							
Are you worried that you could get MERS-CoV infection?	261	No	45	17.2	216	82.8	74.7 (43.5)
Are you worried of getting infected by a corona virus?	582	Yes	460	79.0	122	21.0	
Practice questions							
How do you prevent yourself from getting the virus while at work?	847	Hand washing, use of PPE, avoiding sick patients, isolating sick patients, use of antibiotics, nutrition, vitamins, and other responses in that line (at least 4 correct answers)	391	46.2	456	53.8	46.2 (17.9)
How often do you wear your PPE while at work?	847	Always	417	49.2	430	50.8	
How often do you change your PPE while at work?	847	Where there is need	282	33.3	565	66.7	
Do you use PPE while at work?	847	Yes	724	85.5	123	14.5	
Who handles your used PPE in your facility?	847	Self, Cleaning staff	790	93.3	57	6.7	
How do you dispose used PPE?	847	General disposal bin	249	29.4	598	70.6	
When do you perform hand hygiene while at work?	847	Before contact with patients, after contact with patients, before handling invasive devices, after contact with bodily fluids or non-intact skin or wounds, after contact with inanimate surfaces, after removing sterile or nonsterile gloves (at least 3 correct answers)	790	93.3	57	6.7	
What do you do when you do not have access to PPE at your workstation?	847	I do not see patients/I do not perform my tasks without PPE	185	21.8	662	78.2	
Risk perception questions							
How often do you get flu like symptoms like fever, cough, sore throat, running nose, generalized, body malaise, headache, difficulty in breathing, dizziness, chest congestion?	847	Always	47	5.5	800	94.5	48.4 (29.3)
Have you visited a health facility with respiratory illness in the last 3 months?	847	Yes	226	26.7	621	73.3	
Did you finish the prescribed medication for flu?	847	Yes	208	24.6	639	75.4	

KAP-RP = Knowledge, Attitudes, Practices, and Risk Perception.

Concern about MERS-CoV was reported by 17.2% of respondents. The survey item assessed worry about MERS-CoV infection without specifying whether the perceived risk related to local acquisition or infection associated with travel or exposure outside Kenya. Key preventive practices such as PPE use were moderately followed, with 85.5% reporting PPE usage and 73.7% wearing PPE when handling flu patients. However, only 49.2% consistently wore PPE at work, and only 33.3% changed PPE as needed. Hand hygiene practices were robust, with 93.3% reporting compliance. Perception of flu-like symptoms was low, with only 5.5% reporting frequent symptoms.

In addition, only 26.7% of HCWs had visited health facilities for respiratory illnesses in the 3 months leading to the study, and about a quarter (24.6%) completed prescribed medication for flu.

Supplemental Table 1 compares KAP-RP cores by participant characteristics. Among the 847 participants, 28.1% had sufficient knowledge, 74.7% had a positive attitude, 25.1% demonstrated good practices, and 26.7% had adequate risk perception scores.

Knowledge scores were significantly higher among individuals age 25–34 years (OR = 2.4; 95% CI: 1.30–4.52; *P* = 0.007), 35–44 years (OR = 3.5; 95% CI: 1.85–7.02; *P* <0.001), and 45–54 years (OR = 2.6; 95% CI: 1.26–5.50; *P* = 0.011), in comparison with those 18–24 years of age. Males had higher odds of sufficient knowledge than females (OR = 1.4; 95% CI: 1.04–1.97; *P* = 0.03). Clinical staff were more than three times more likely to have sufficient knowledge in comparison with nonclinical staff (OR = 3.2; 95% CI: 2.28–4.57; *P* <0.001). County and residence were not significantly associated with knowledge scores.

Older age groups (25–34, 35–44, and 45–54 years) had significantly lower odds of sufficient attitude in comparison with 18- to 24-year-olds. Clinical staff were less likely to have a sufficient attitude (OR = 0.3; 95% CI: 0.23–0.47; *P* <0.001). Sex and residence were not significantly associated with attitude.

Males were less likely to have good practices than females (OR = 0.66; 95% CI: 0.48–0.92; *P* = 0.013). Clinical staff had better practice scores than nonclinical staff (OR = 1.48; 95% CI: 1.06–2.09; *P* = 0.024), and participants from Marsabit were significantly less likely to practice appropriate measures in comparison with those from Isiolo (OR = 0.37; 95% CI: 0.26–0.53; *P* <0.001).

**Figure 3.**
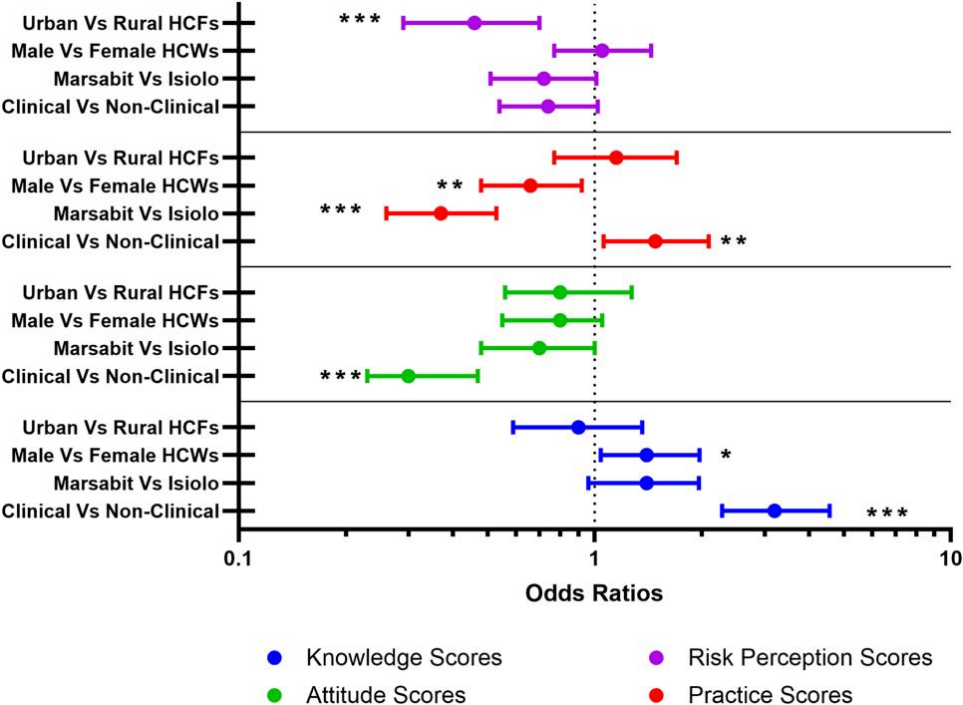
Graph comparing KAP-RP scores among health care workers by demographic categories.

Last, for risk perception, urban residents had significantly lower odds of sufficient risk perception than rural counterparts (OR = 0.46; 95% CI: 0.29–0.70; *P* <0.001). Clinical staff had marginally lower odds of sufficient risk perception in comparison with nonclinical staff (OR = 0.74; 95% CI: 0.54–1.02; *P* = 0.067). No significant associations were observed between risk perception and sex or age. A graphical comparison of KAP-RP scores across respondent characteristics is presented in Figure 3.

Table 3 presents the results of a Pearson’s correlation analysis examining the relationships among risk perception, practice, attitude (worry), and knowledge scores. The results indicated that the correlation between risk perception and other variables, namely practice (*r* = 0.026, *P* = 0.453), attitude (*r* = –0.053; *P* = 0.122), and knowledge (*r* = 0.060; *P* = 0.083) was weak and not statistically significant.

**Table 3:** Correlation analysis of KAP-RP scores

Variable	Pearson’s Correlation Coefficient	95% CI	*P*-Value
Risk – Practice	0.03	(−0.04 to 0.1)	0.372
Risk – Attitude	−0.05	(−0.12 to 0.01)	0.122
Risk – Knowledge	0.06	(−0.01 to 0.13)	0.083
Practice – Attitude	−0.11	(−0.18 to −0.05)*	**0.001**
Practice – Knowledge	0.11	(0.04 to 0.18)*	**0.002**
Attitude – Knowledge	−0.83	(−0.85 to −0.81)*	**<0.001**

KAP-RP = Knowledge, Attitudes, Practices, and Risk Perception.

* Significant correlation at *P* <0.05 (two-tailed).

In contrast, significant associations were observed between other variable pairs. There was a weak but statistically significant negative correlation between practice and attitude (*r* = –0.113; *P* = 0.001), suggesting that increased worry was modestly associated with lower practice scores. Similarly, a positive correlation was found between practice and knowledge (*r* = 0.106; *P* = 0.002), indicating that better knowledge was associated with improved practices.

The most notable finding was a very strong and statistically significant negative correlation between attitude (worry) and knowledge (*r* = –0.829; *P* <0.001), demonstrating that higher levels of worry were strongly linked to lower knowledge scores.

## DISCUSSION

Despite the heightened global awareness of coronaviruses after the COVID-19 pandemic, our study found that HCWs working in high camel–density regions demonstrated low (<30%) knowledge and poor risk perception of MERS-CoV. In the absence of reported human cases in Kenya and prior to any formal MERS-CoV–specific sensitization or training, this finding reflects an expected baseline level of awareness rather than a deficiency in individual competence. Notably, no significant differences were observed between clinical and nonclinical staff in knowledge or risk perception, and fewer than 30% of participants reported adequate adherence to infection prevention practices. Collectively, these findings point to a system-level preparedness gap in a high-risk zoonotic setting and contrast markedly with reports from MERS-endemic regions and high-income countries, underscoring the need for proactive preparedness strategies in resource-limited settings such as northern Kenya, where MERS-CoV is known to circulate in camels.

Our finding of 28.1% sufficient knowledge among Kenyan HCWs is substantially lower than reported rates from Saudi Arabia, the epicenter of MERS-CoV outbreaks. Studies from Saudi health care facilities have documented knowledge adequacy rates ranging from 32.4% to >79%, with most falling between 50% and 70%.^13^^,^^19^^,^^20^ In southwestern Saudi Arabia, 51% of HCWs demonstrated sufficient MERS-CoV knowledge, whereas a national survey in Makkah hospitals found 32.4% with good knowledge scores.^21^ The disparity becomes more pronounced when comparing with developed countries for emerging infectious diseases. HCWs in high-resource settings typically demonstrate 70% to 85% knowledge adequacy for epidemic-prone diseases, supported by robust infection control infrastructure, regular training programs, and established surveillance systems.^22^^,^^23^ The stark contrast could suggest that proximity to the outbreak epicenter and direct exposure to MERS-CoV cases significantly influence health systems and hence HCW preparedness, whereas peripheral regions with zoonotic risk remain inadequately prepared despite their vulnerability. This gap could be pointing to a lack of emphasis on these aspects in outbreak preparedness.

Our study revealed complex rural–urban differences that both align with and diverge from established patterns in the literature. Although 77% of our participants worked in rural facilities, we found that urban residents had significantly lower odds of sufficient risk, contrasting with typical expectations that urban HCWs would demonstrate superior knowledge and practices. This finding diverges from documented patterns in sub-Saharan Africa, where urban areas generally have better health care outcomes and resources, though specific comparative data on HCW knowledge remain limited.^24^^,^^25^ The reversed pattern in our study may reflect unique contextual factors: rural HCWs in northern Kenya have greater exposure to camel-related health risks and may perceive higher personal vulnerability, leading to heightened risk awareness despite knowledge gaps. At the same time, heightened risk perception among rural staff may reflect concerns arising from constrained infection prevention infrastructure, limited availability of PPE, and logistical challenges in rural facilities. These findings underscore that preparedness gaps extend beyond individual HCW knowledge to broader health-system readiness, highlighting the importance of strengthening infection prevention capacity, supply chains, community risk communication, and One Health collaboration with the animal health sector in zoonotic hot spot settings.

However, our findings align with rural health care challenges documented across developing countries. The low practice adequacy rate (25.1%) mirrors patterns seen in rural African health care settings, where infrastructure limitations, supply chain disruptions, and resource constraints significantly impede infection control practice implementation.^26^^,^^27^ Studies from other East African countries, particularly Ethiopia, report similar practice–knowledge gaps, where HCWs possess theoretical understanding but face systemic barriers to implementation.^27^^,^^28^

One of our most striking findings was the strong negative correlation between knowledge and attitude, indicating that higher knowledge was associated with lower worry about coronavirus infections. This paradoxical relationship challenges conventional health behavior models that assume knowledge leads to appropriate risk perception and protective behaviors. This finding contrasts with studies from Saudi Arabia, where knowledge and positive attitudes typically showed positive correlations.^29^^,^^30^ In developed countries, the knowledge–attitude relationship is generally positive but weaker (*r* = 0.2 to 0.4), suggesting that other factors, such as institutional support and professional confidence, mediate this relationship.^31^^,^^32^

The inverse relationship observed in our study may reflect several contextual factors unique to resource-limited settings with endemic zoonotic risks. HCWs with greater knowledge may develop a false sense of security, believing their understanding provides adequate protection despite inadequate resources for implementation. Alternatively, increased knowledge may reveal the limitations of available protective measures, leading to resignation rather than heightened concern. This pattern has been observed in other resource-constrained settings facing endemic infectious disease threats.^33^^,^^34^

Our findings of inadequate knowledge (28.1%), poor practices (25.1%), and low risk perception (26.7%) among HCWs in this zoonotic hot spot represent a unsustainable preparedness gap. Should a more virulent or transmissible MERS-CoV variant emerge, health care facilities in northern Kenya with their limited infection-control infrastructure and inadequately prepared workforce could become amplification sites for regional and global spread. The COVID-19 pandemic demonstrated how health care–associated transmission can rapidly overwhelm health systems; a MERS-CoV pandemic with its higher mortality rate could prove even more devastating in settings where frontline workers lack adequate knowledge and protective practices.

### Limitations and future directions.

Our study has several limitations that should be considered when interpreting these findings. The cross-sectional design precludes causal inference, and self-reported practice measures may overestimate actual adherence to infection-control protocols. In addition, the study was conducted during the post–COVID-19 period, which may have influenced HCWs’ perceptions and responses regarding coronavirus infections. The survey did not explicitly distinguish between perceptions of locally acquired MERS-CoV infection and concern related to infection in other geographic settings, limiting our ability to determine whether reported worry reflected perceived local occupational risk.

Future research should use longitudinal designs to track changes in KAP over time and include objective measures of infection-control practice adherence. Incorporating survey items that explicitly assess beliefs about local zoonotic transmission, non–travel-related human infection, and routine testing practices would strengthen future assessments. Mixed-methods approaches could provide deeper insights into the contextual factors driving the observed knowledge–attitude paradox and help identify culturally appropriate intervention strategies.

## CONCLUSION

This study reveals substantial gaps in MERS-CoV knowledge, risk perception, and infection-control practices among HCWs in northern Kenya, with performance levels well below those reported from endemic regions and developed countries. The inverse relationship between knowledge and attitude, combined with weak knowledge–practice correlations, suggests that conventional education-based interventions may be insufficient for improving preparedness in resource-limited zoonotic hot spots.

Context-specific interventions that address not only knowledge gaps but also structural barriers, cultural factors, and motivational drivers are urgently needed to enhance infection prevention and preparedness among frontline health workers in high-risk regions. The findings underscore the critical need for tailored approaches that consider the unique challenges facing health care systems in zoonotic disease hot spots, moving beyond traditional knowledge transfer to comprehensive capacity building that addresses systemic barriers to effective infection control.

## Supplemental Materials

10.4269/ajtmh.25-0585 Supplemental Materials
