# Supplementary material for: Knowledge, Attitudes, Practices, and Risk Perception of MERS-CoV and Other Coronaviruses Among Health Care Workers in Northern Kenya
Source: Am J Trop Med Hyg. 2026 May 12;115(1):153–61. doi: 10.4269/ajtmh.25-0585 (PMC13326877; doi:10.4269/ajtmh.25-0585)
Supplement: Supplemental Materials [file tpmd250585.SD1.pdf]

Supplemental Table 1

Comparison of Knowledge, Attitude, Practices, and Risk Perception Scores by respondent characteristics

Characteristics	Knowledge Scores							
		Insufficient		Sufficient		OR	95% CI	*P*-Value
	N	*n*	(%)	*n*	(%)			
	**847**	**609**	**71.9**	**238**	**28.1**			
**Age categories**								
18 to 24	96	81	84.4	15	15.6	Ref.	Ref.	Ref.
25 to 34	409	285	69.7	124	30.3	2.4	1.30, 4.52	0.007
35 to 44	185	122	65.9	63	34.1	3.5	1.85, 7.02	<0.001
45 to 54	109	80	73.4	29	26.6	2.6	1.26, 5.50	0.011
55 and above	48	41	85.4	7	14.6	1.4	0.48, 3.72	0.5
**Sex**								
Female	373	283	75.9	90	24.1	Ref.	Ref.	Ref.
Male	474	326	68.8	148	31.2	1.4	1.04, 1.97	0.03
**Cadre**								
Non-clinical[1]	371	309	83.3	62	16.7	Ref.	Ref.	Ref.
Clinical[2]	476	300	63.0	176	37.0	3.2	2.28, 4.57	<0.001
**County**								
Isiolo	308	233	75.6	75	24.4	Ref.	Ref.	Ref.
Marsabit	539	376	69.8	163	30.2	1.4	0.96, 1.96	0.081
**Residence**								
Rural[3]	652	461	70.7	191	29.3	Ref.	Ref.	Ref.
Urban[4]	195	148	75.9	47	24.1	0.9	0.59, 1.36	0.6
	**Attitude Score**							
		**Insufficient**		**Sufficient**		**OR**	**95% CI**	**P-Value**
	N	***n***	**(%)**	***n***	**(%)**			
	**847**	**214**	**25.3**	**633**	**74.7**			
**Age categories**								
18 to 24	96	15	15.6	81	84.4	Ref.	Ref.	Ref.
25 to 34	409	110	26.9	299	73.1	0.5	0.24, 0.86	0.018
35 to 44	185	54	29.2	131	70.8	0.3	0.17, 0.66	0.002
45 to 54	109	27	24.8	82	75.2	0.4	0.19, 0.84	0.017
55 and above	48	8	16.7	40	83.3	0.6	0.22, 1.60	0.3
**Sex**								
Female	373	83	22.3	290	77.7	Ref.	Ref.	Ref.
Male	474	131	27.6	343	72.4	0.8	0.55, 1.05	0.1
**Cadre**								
Non-clinical[1]	371	56	15.1	315	84.9	Ref.	Ref.	Ref.

Clinical[2]	476	158	33.2	318	66.8	0.3	0.23, 0.47	<0.001
**County**								
Isiolo	308	68	22.1	240	77.9	Ref.	Ref.	Ref.
Marsabit	539	146	27.1	393	72.9	0.7	0.48, 1.00	0.05
**Residence**								
Rural[3]	652	165	25.3	487	74.7	Ref.	Ref.	Ref.
Urban[4]	195	49	25.1	146	74.9	0.8	0.56, 1.27	0.4
	**Practices Score**							
		**Insufficient**		**Sufficient**		**OR**	**95% CI**	**P-Value**
	N	***n***	**(%)**	***n***	**(%)**			
	**847**	**634**	**74.9**	**213**	**25.1**			
**Age categories**								
18 to 24	96	67	69.8	29	30.2	Ref.	Ref.	Ref.
25 to 34	409	313	76.5	96	23.5	1.01	0.60, 1.73	>0.9
35 to 44	185	130	70.3	55	29.7	1.35	0.77, 2.42	0.3
45 to 54	109	80	73.4	29	26.6	1.23	0.64, 2.35	0.5
55 and above	48	44	91.7	4	8.3	0.30	0.08, 0.87	0.040
**Sex**								
Female	373	266	71.3	107	28.7	Ref.	Ref.	Ref.
Male	474	368	77.6	106	22.4	0.66	0.48, 0.92	0.013
**Cadre**								
Non-clinical[1]	371	295	79.5	76	20.5	Ref.	Ref.	Ref.
Clinical[2]	476	339	71.2	137	28.8	1.48	1.06, 2.09	0.024
**County**								
Isiolo	308	192	62.3	116	37.7	Ref.	Ref.	Ref.
Marsabit	539	442	82.0	97	18.0	0.37	0.26, 0.53	<0.001
**Residence**								
Rural[3]	652	504	77.3	148	22.7	Ref.	Ref.	Ref.
Urban[4]	195	130	66.7	65	33.3	1.15	0.77, 1.70	0.5
	**Risk Perception Scores**							
		**Insufficient**		**Sufficient**		**OR**	**95% CI**	**P-Value**
	N	***n***	**(%)**	***n***	**(%)**			
	**847**	**621**	**73.3**	**226**	**26.7**			
**Age categories**								
18 to 24	96	74	77.1	22	22.9	Ref.	Ref.	Ref.
25 to 34	409	297	72.6	112	27.4	1.08	0.63, 1.89	0.8
35 to 44	185	136	73.5	49	26.5	0.99	0.55, 1.83	>0.9
45 to 54	109	78	71.6	31	28.4	1.09	0.57, 2.11	0.8
55 and above	48	36	75	12	25	0.86	0.36, 1.96	0.7
**Sex**								
Female	373	275	73.7	98	26.3	Ref.	Ref.	Ref.
Male	474	346	73	128	27	1.05	0.77, 1.44	0.8
**Cadre**								

Non-clinical[1]	371	260	70.1	111	29.9	Ref.	Ref.	Ref.
Clinical[2]	476	361	75.8	115	24.2	0.74	0.54, 1.02	0.067
**County**								
Isiolo	**308**	**223**	**72.4**	**85**	**27.6**	**Ref.**	**Ref.**	**Ref.**
Marsabit	539	398	73.8	141	26.2	0.72	0.51, 1.01	0.055
**Residence**								
Rural[3]	652	460	70.6	192	29.4	Ref.	Ref.	Ref.
Urban[4]	195	161	82.6	34	17.4	0.46	0.29, 0.70	<0.001

1: Accounts, Board Member, Casuality, Link assistant, Manager, Storekeeper, Administrative, Administrator, Cleaner, Clerk, Cook, Data, Driver, Front office/customer care/secretary, Gardener, Grounds, Health, Health assistant, Housekeeper, Human resource, ICT, Interpreter, Laundry, Link assistants, Maintenance personnel, Medical engineer, Mentor, Mortuary, Public, Record's officer, Records officer, Renal Unit, Security, Social, Social worker

***2:** Clinical Officer, Community Health Volunteer, Dentist, Doctor (Consultant, medical officer), Intern, Laboratory technologist, Nurse, Nurse aide / Nurse assistant, Nutritionist, Occupational, Orthopedic, Pharmacist / Pharmacy technologist, Physiotherapist, Sonographer, Student, Theatre

***3:** Urban: Marsabit County Referral Hospital, Moyale Sub-County Referral Hospital, and Isiolo County Referral and Teaching Hospital

***4:** Rural: None Referral Hospitals
